# The frequency of *Klebsiella pneumonia* encoding genes for CTX-M, TEM-1 and SHV-1 extended-spectrum beta lactamases enzymes isolated from urinary tract infection

**DOI:** 10.1186/s12941-018-0256-y

**Published:** 2018-02-13

**Authors:** Masomeh Dehshiri, Seyed Sajjad Khoramrooz, Mohammad Zoladl, Seyed Abdolmajid Khosravani, Najmeh Parhizgari, Mohammad Hossein Motazedian, Soheyla Jahedi, Asghar Sharifi

**Affiliations:** 1 Cellular and Molecular Research Centre, Yasuj University of Medical Sciences, Yasuj, Iran; 2 Department of Parasitology and Mycology, Shiraz University of Medical Sciences, Shiraz, Iran; 3 Paradise shahid Bahonar, Farhangian University of Shiraz, Shiraz, Iran

**Keywords:** Antibiotic resistance, Extended-spectrum β-lactamase, *K. pneumonia*, Urinary tract infection, Genes SHV, TEM, CTXM

## Abstract

**Background:**

The extended- spectrum β-lactamase producing bacteria are widely spread worldwide. The productions of these enzymes cause bacterial resistance to a wide range of antibiotics. The aim of this study was to investigated the frequency of *K. pneumonia* encoding genes for CTX-M, TEM-1 and SHV-1 extended-spectrum beta lactamases enzymes isolated from urinary tract infection.

**Methods:**

This study is cross-sectional study. All *K. pneumonia* isolates from urine samples, which had grown on media culture more than 105 were delivered to the medical microbiology laboratory. *K. pneumonia* susceptibility of 198 samples were confirmed by disk diffusion. The gene frequency of genes was determined using PCR, and analyzed using SPSS version 21 software.

**Finding:**

Most of the *K. pneumonia* isolated from urine producing β-lactamase were resistant to cotrimoxazole (53.2%) followed by cefotaxime (50%), ceftazidime, ceftriaxone (40.3%), nalidixic acid (17.8%), amikacin and imipenem (1.6%) and meropenem (0%) respectively. Out of the 198 confirmed isolates of *K. pneumonia*, 62 cases (31.3%) have the gene phenotype of broad spectrum β-lactamase enzymes and highest frequency of gene phenotype was related to the SHV-1 gene (85.5%). Then in the terms of abundance from highest to lowest CTXM-3 (56.5%), CTXM-1 (27.4%), TEM-1 (16.1%) and CTXM-2 (8.1%), were respectively.

**Conclusion:**

This study showed that *K. pneumonia* isolated from urine producing β-lactamase were resistance to a wide range of antibiotics. Due to the increasing resistance of most antibiotics, control and supervision in the use of antibiotics and identification of broad spectrum β-lactamase enzymes by phenotypic methods appears to be essential.

## Background

Klebsiella is a Gram negative, non-motile, facultative anaerobic, catalase positive, oxidase negative, and usually encapsulated bacteria. It ferments sugars such as lactose and sucrose. Most strains produce gas from fermentation and starch which is an important distinguishing feature of these bacteria. The bacteria are part of Enterobacteriaceae family and *K. pneumonia* strain is an indicator species of this genus of bacteria [1]. *K. pneumonia* is an opportunistic pathogen among enterobacteriacae family, Klebsiella The genus Klebsiella has two pathogenic species; *K. pneumonia* and *K. oxytoka* [2].

*Klebsiella pneumonia* is closely related to *K. oxytoka*, and recognized from the latter by being indole negative and its growth ability on 3-hydroxybutyrate [2, 3]. This bacterium is available in gasterointestinal, eye, respiratory, and urogenital tract in healthy human. It exists as saprophyte microorganisms in nasopharynx of 1–6% individuals. The prevalence increases up to 20% in patient who are admitted in hospitals. Infections are less common outside the hospitals, and routes of transmission are usually hospital staff hands, antiseptic solutions, working containers, and equipments [4]. Anesthesia and artificial respiration instruments are the best means of infection transport due to dampness. In general, patients over 40 years old those with immunodeficiency syndromes, and underlying medical conditions such as diabetes mellitus and chronic lung disease are the objective of this bacterium [4, 5].

β-Lactamases from TEM type are often found in *K. pneumonia*. These enzymes are derived from TEM-1 and TEM-2 enzymes. TEM-1 is the most important β-lactamase in Gram negative bacteria. Both TEM-1 and TEM-2 are capable of hydrolyzing penicillin and first generation cephalosporin but are not able to invade oxyimino–cephalosporin. Amino acid replacement can be created in many places of TEM-1. β-lactamase in vitro without losing the most activity. These changes are responsible for phenotypic extended spectrum β-lactamases (ESBL) change and enzyme activity site that gives access to oxy Mino β-lactamase. More than 130 common TEM enzymes have been identified, which provide a useful method for the distribution of resistant genes [6, 7].

### β-Lactamase SHV type (class A)

These enzymes are derived from SHV-1. They are the first enzymes of SHV class which are found in *K. pneumonia* and are responsible for 20% of ampicillin resistance in the present samples [8, 9].

There exists a structural likeness between SHV-1 and TEM-1 in general, and a 68% amino acid similarity. More than 50 SHV varieties have come to be known based on the replacement of amino acid combinations.

### β-Lactamases CTX-M type (class A)

CTX is representative of the high hydrolysis power of β-lactamase against cefotaxime. More than 40 CTX-M enzymes are known so far, which can be classified in five groups.

1. CTX-M group which contains 6 enzymes dependent on plasmid.
  A-CTX-M1, CTX-M3, CTX-M-10, CTX-M-12, CTX_M-15 and, FEC-4.
2. CTX-M-2 group which contains 8 CTX-M enzyme dependent on plasmid.
  CTX-M-2, CTX-M-4, CTX-M-4L, CTX-M-5, CTX-M-6, CTX-M-7, CTX-M-20 and Toto-1.
3. CTX-M-8 group which contains 1 member dependent on plasmid.
4. CTX-M-9 group which contains 9 enzymes dependent on plasmid.
  CTX-M-9, CTX-M-13, CTX-M-14, CTX-M-16, CTX-M-17, CTX-M-19, CTX-M-21, CTX-M-27 and Toho 2.
5. CTX-M-25 group containing CTX-M-25 and CTX-M-26, CTX-M-2, CTX-M-3 and CTX-M-14 which have the most dispersion (3, 32, 52, 53).

These enzymes are capable of cephalothin hydrolysis better than penicillin and cefaloxine, and more than Ceftazidime. CTXM type is predominantly found in three geographic areas; South America, The Far East, and Europe [10, 11].

The aim of present study was to indicate the frequency of Klebsiella isolates encoding SHV-1, TEM-1, CTXM genes and the broad spectrum β-lactamase enzymes in urinary tract infection (UTI) patients in Yasuj City, Kohgiluyeh and Boyer-Ahmad province, Iran, during 2014–2015.

## Methods

This study sought to determine the prevalence of *K. pneumonia* isolates encoding SHV-1, TEM-1, CTXM genes and the broad spectrum β-lactamase enzymes in UTI patients in Yasuj City during 2014–2015. In this sense, statistical, cross sectional, and descriptive analysis methods were employed.

### Ethics approval and consent to participate

Ethics approval (MEC: 93.05.06.07) was obtained prior to the start of this study.

### Sampling and culture methods

This study was carried out during October 2014 to August 2015. Urinary samples of patients confirmed with UTI from three laboratories around the city were sent to microbiology laboratory of Yasuj University of Medical Science. Then, each sample was cultured on EMB media. Further diagnostic tests followed as described below:

#### Phenotyping test for ESBL production

 Confirmatory tests for ESBL production based on CLSI standards were carried out on the bacteria. First, bacteria were inoculated on MHA media culture containing ceftazidime and cefotaxime disks. Both plates were inoculated alike and kept on incubator for 18–24 h. The zone of inhibition diameter (ZOID) was measured around each disk and ZOIDs more than 5 mm were considered positive [12].

#### Polymerase chain reaction (PCR)

Bacterial genome extraction by boiling method: One colony from fresh bacteria was solved in 300 ml distilled water and homogenized with vortex and kept on thermo block in 100° C for 10 min, and then centrifuged in 12,000 rpm for 10 min and the supernatant containing genome was used for PCR [13].

## Mastermix preparation

Five micro litters of purified DNA, 1 µl FORWARD primer (20 pmol), 1 µl REVERSE primer (20 µmol), and 12.5 µl mastermixe were mixed and the volume was brought to 25 µl by adding distilled water.

Primary denaturation (92 °C for 2 min), 30 thermal cycles of denaturation (95 °C for 1 min), primer binding (57 °C for 30 s for β-lactamase SHV gene), 61 °C for 20 s for β-lactamase TEM gene, 55 °C for 30 s for β-lactamase CTXM-1, 55 °C for 30 s for β-lactamase CTX M2 gene, 59 °C for 20 s for bla CTXM2 gene, proliferation temperature 72 °C for 30 s, and final proliferation temperature (72 °C for 5 min) were performed [14].

Samples were put into gel wells which had already been prepared. Then the electrodes were connected (red wire was the sign of positive electric charge which was placed in the bottom and black wire was the sign of negative electric charge sign which was placed next to gel holes). After the connection of electrodes, the instrument was adjusted to 100 voltage for 1 h with 700 mA flow (current strength), and then turned on.

### Photography from gel

The gel was placed inside the device and then the device was turned on with the correct setting (Table 1).

**Table 1:** Primers used for ESBL genes presence in *Klebsiella*

Gene name	Primer sequence 5–3	Product size (bp)
SHV	F:5′ TCAGCGAAAAACACCTTG 3′	472
	R:5′ TCCCGCAGATAAATCACC 3′	
TEM	F: 5′ CTTCCTGTTTTTGCTCACC 3′	632
	R: 5′ AGCAATAAACCAGCCAGC 3′	
CTXM-1	F: 5′ GAC GAT GTC ACT GGC TGA GC 3′	499
	R: 5′ AGC CG C CGA CGC TAA TAC A 3′	
CTXM2	F: 5′ GCG ACC TGG TTA ACT ACA ATCC 3′	351
	R: 5′ CGG TAG TAT TGC CCT TAA GCC 3′	
CTXM3	F: 5′ CGC TTT GCC ATG TGC AGC ACC 3′	307
	R: 5′ GCT CAG TAC GAT CGA GCC 3′	

### Data analysis method

For data analysis, SPSS software version 21 was applied. Both descriptive statistics such as frequency, mean, standard deviation, graph, and statistical inferential tests such as Chi square, fishers exact test, independent T test, and one-way variance analytic test were used. It is worth noting that in this study, before inferential statistical tests with Kolmogorov–Smirnov test, variable study from normal distribution was reviewed and approved.

## Finding

Out of 986 samples suspicious to *K. pneumonia* UTI, 198 (20.1%) were confirmed based on Klebsiella diagnostic test. The results are summarized in Table 2.

**Table 2:** Frequency distribution *Klebsiella pneumoniae* isolated from UTI patients referred to clinical laboratories according to Gender in Yasuj city

Confirmed tests for K.p on samples urine sample according to gender	Sum		Non confirmed		Confirmed	
	Percent	Number	Percent	Number	Percent	Number
Male	48.4	477	42.3	417	6.1	60
Female	51.6	509	37.6	371	14	138
Sum	100	986	79.9	788	20.1	198

Based on Table 2 out of 198 samples, 138 were female patients and 60 were males. The minimum and maximum ages were 1 month to 68 years respectively with the standard deviation of 16.33 ± 17.92.

### Susceptibility test of *K. pneumonia* to CLSI antibiotics

*Klebsiella pneumonia* in vitro susceptibility test against antibiotics such as Amikacin (AMK 30 mg), Ceftriaxone (CTK 30 mg), Meropenem (MRP 10 mg), Nalidixic acid (NAL 30 mg), Cefotaxim (CTX 30 mg), Ceftazidime (CAZ 30 mg), Cefotaxime (CTX + C 40 mg), and Ceftazidime (CAZ + C 40 mg) were examined Table 3.

**Table 3:** Frequency distribution of *Klebsiella pneumoniae* isolation according to resistant to antibiotics

Antibiotic results	Sensitive		Semisensitive		Resistance		Total	
Antibiotic name	Percent	Number	Percent	Number	Percent	Number	Percent	Number
Amikacin	98	194	1	2	1	2	100	198
Ceftriaxon	86	170	1.5	3	12.5	25	100	198
Co-trimoxazol	71	141	1.5	3	27.5	54	100	198
Imipenem	99	196	0	0	1	2	100	198
Meropenem	100	198	0	0	0	0	100	198
Nalidixic acid	82.5	163	8.5	17	9	18	100	198
Cefotaxim	84	166	0.5	1	15.5	31	100	198
Cefazidim	84.5	167	3	6	12.5	25	100	198

Based on these findings, the maximum resistance of *K. pneumonia* isolates was against Co-trimoxazole (27.5%). The remaining resistance intensities from highest to lowest were as follows: Cefotaxime (15.5%), Ceftazidin, Ceftriaxone (12.5%), Nalidixic acid (9%), Amikacin, Imipenem (1%) and Meropenem (0%).

Frequency distribution of *K. pneumonia* isolates according to broad spectrum β-lactamase phenotype and sex are revealed in Table 4.

**Table 4:** Frequency distribution of *Klebsiella pneumoniae* according to broad spectrum β-lactamase phenotype and sex

Broad spectrum β-lactamase phenotype from urine samples according to sex	ESBL+		ESBL−		Sum	
	Percent	Number	Percent	Number	Percent	Number
Male	13.6	27	16.7	33	30.3	60
Female	17.7	35	52	103	69.7	138
Sum	31.3	62	68.7	136	100	198

**Fig. 1:**
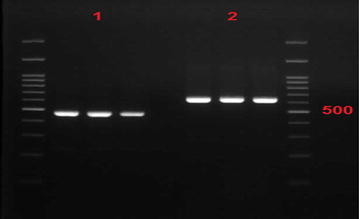
Electrophoresis gel sample SHV-1 and TEM-1 in *Klebsiella pneumoniae* isolates producing broad spectrum β-lactamase (ESBL). Column No. 1 has SHV-1 with size band 472 and column No. 2 has TEM-1 gene with band size 632

**Fig. 2:**
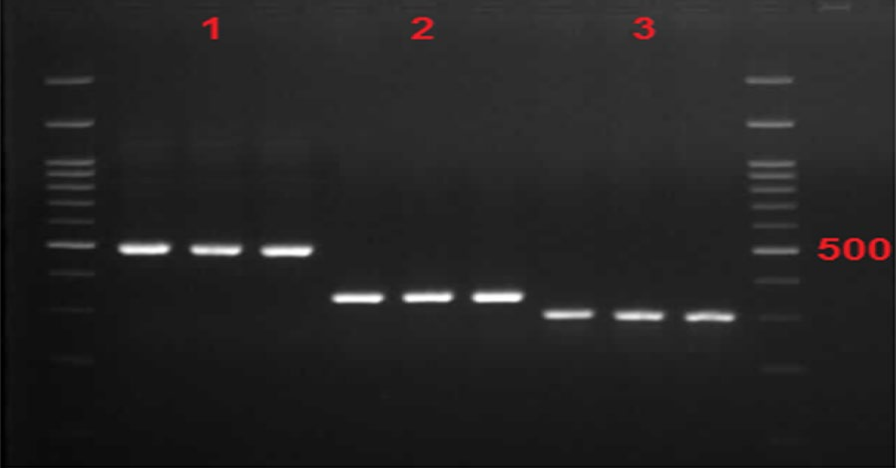
Electrophoresis gel sample confirming CTXM-1, CTXM-2, and CTXM gene in *Klebsiella pneumoniae* isolation producing ESBL In this figure, column No. 1 belongs to CTXM-1 with 499 band size, column No II has CTXM-2 genes with 351 band size, and column No. III has CTXM-3 with 307 band size

**Fig. 3:**
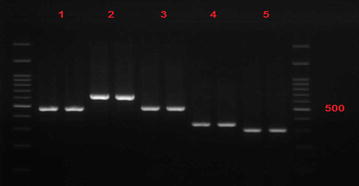
Frequency distribution of *B. lactamase* genes TEM-1, SHV-1, CTXM-1, CTXM-2, and CTXM-3 in *Klebsiella pneumoniae* isolates producing ESBL Column No.1 has SHV-1 gene with 472 band size, column No. 2 has TEM-1 gene with 632 band size, column No. III has CTXM-1 with 499 band size, column No. III has CTXM-2 gene with 351 band size, and column No. 5 has CTXM-3 with 307 band size

Frequency of β-lactamase genes TEM-1, SHV-1, CTXM-2, and CTXM-3 in 62 samples producing ESBL was estimated by PCR and shown in Figs. 1, 2, 3 and Table 5. As noticed, the most synchronizing pattern belonged to B lactamase genes SHV-1 and CTXM-3 (30.7%).

**Table 5:** Frequency of β-lactamase genes synchronization in *Klebsiella pneumoniae* strains of producing ESBL

β-Lactamase pattern genes in K.p producing ESBL	Relative frequency %	Number absolute frequency
Wihtout β-lactamase	8.1	5
SHV-1 gene alone	24.3	15
CTXM-3 gene alone	4.8	3
β-Lactamase genes(SHV-1, CTXM-1 and CTXM-2) synchronization	6.4	4
β-Lactamase genes(SHV-1 and CTXM-3) synchronization	30.7	19
β-Lactamase genes(TEM-1, SHV-1, CTXM-1 and CTXM-3) synchronization	9.7	6
β-Lactamase genes(SHV-1, CTXM-1, CTXM-2 and CTXM-3) synchronization	1.6	1
β-Lactamase genes(CTXM-1 and CTXM-3) synchronization	1.6	1
β-Lactamase genes(SHV-1 and, CTXM-) synchronization	3.2	2
β-Lactamase genes(TEM-1, SHV-1, CTXM-1, CTXM-2 and CTXM-3) synchronization	1.6	1
β-Lactamase genes(SHV-1, CTXM-1 and CTXM-2) synchronization	1.6	1
β-Lactamase genes(TEM-1, SHV-1, CTXM-1 and CTXM-2) synchronization	1.6	1
β-Lactamase genes(TEM-1 and SHV-1) synchronization	3.2	2
β-Lactamase genes(SHV-1, CTXM-1 and CTXM-3) synchronization	1.6	1
Sum	100	62

All *K. pneumonia* isolates were susceptible to meropenem and most antibiotic resistance in both genders were observed with Co-trimoxazole. No statistically significant difference was seen between antibiotic resistance of isolates by gender or age. Also, presence of β-lactamase genes such as TEM-1, SHV-1,CYXM-2, and CTXM-3 confirmed with PCR. No statistically significantly differ was observed among individuals of various ages. As Table 6 showed the most commonly ESBL producing gene was belonged to SHV-1(85.5%), followed by highest to lowest CTXM-3(56.5%), CTXM-1(27.4%), TEM-1(16.1%) and CTXM-2(8.1%) respectively.

**Table 6:** Frequency distribution β-lactamase genes (TEM-1,SHV-1,CTXM-1, CTXM-2 and CTXM-3) *Klebsiella pneumoniae* strains producing broad spectrum ESBL genes

Presence of K.p genes strain situation producing ESBL	Having		Not having		Sum	
Gene name	Percent	Number	Percent	Number	Percent	Number
β-Lactamase gene TEM-1	16.1	10	83.9	52	100	62
β-Lactamase gene SHV-1	85.5	53	14.5	9	100	62
β-Lactamase gene CTXM-1	27.4	17	72.6	45	100	62
β-Lactamase gene CTXM-2	8.1	5	91.9	57	100	62
β-Lactamase gene CTXM-3	56.5	35	43.5	27	100	62
β-Lactamase gene TEM-1	16.1	10	83.9	52	100	62

## Discussion

*Klebsiella pneumonia* is a common cause of nosocomial infections such as UTI. The importance of UTI is that if not diagnosed or diagnosed too late it will lead to renal failure.

β-Lactam drugs are one of the most effective drugs in UTI treatment. The ESBL bacteria, with inactivation of a wide range of β-lactam drugs especially cephalosporin and monobactam, cause treatment failure and increase healthcare costs. Therefore, the study of gene resistance to β-lactamase is important. It seems that the emergence and spread of these bacteria are due to prolonged hospitalization, increased consumption of β-lactam antibiotics (especially ceftazidime), use of catheters, and experimental treatments against antibiotic-resistant [16].

The aim of this study was to determine the prevalence of *K. pneumonia* producing ESBL and molecular diagnosis of B lactamase genes such as β-lactamase CTX-M, β-lactamase TEM, and β-lactamase SHV).

In present study out of 198 samples, 62 (31.3%) were ESBL productive (27 separated from males and 35 from females). Published result from different scientific researches related to ESBL production in Iran is very diverse ranging from 9.8% to 75.7% depending on infection control system and therapeutic regimens. Another study from Bazaz et al. reported the prevalence of ESBLs in *K. pneumonia* and *E. coli* bacteria as high as 59.2%. In another research on 218 bacterial strains, 70 (32%) strains of Klebsiella and 49 (70%) strains of *K. pneumonia* contained ESBL [13]. Reports on ESBL production rate in *K. pneumonia* isolates from different countries also show significant variations. In a study in Japan and USA the prevalence rate was reported 40 and 44% respectively. However, in Southeast Asia, the prevalence rates of ESBL reported varied from 20% to more than 60%. ESBL production in India, Taiwan, Thailand, Pakistan, and Korea were reported 44, 8–29, 64, 36–56 and 22% respectively [14, 15].

The prevalence rate of ESBL production was reported by Ajas et al. in Lahore Pakistan 71% [16].

Due to emergence of bacterial resistance and ESBL production In a region compared to other regions is different. Also, the treatments of this infection with cephalosporins and other broad-spectrum antimicrobials have led to the emergence of multi-drug resistance. In this study, bacterial resistance was shown to be 30.4, 50, and 61.1% respectively in the case of cephalosporin-ceftriaxone and imipenem.

Mullana et al. demonstrated that the resistance of *K. pneumonia* strains in clinical samples of Babol city was against ceftriaxone (60%) cefotaxime (90%), and imipenem (60%) [17].

In the present study resistance to amikacin was 61.1%, ceftazidime 32.40%, nalidixic acid 74.17% and sensitivity to imipenem was 38.98%.

Ishim in Japan determined that the imipenem as an effective antibiotic for *K. pneumonia* treatment [18].

PCR results in our research on β-lactamase Drug resistance to antibiotics in different regions of Iran and worldwide is differs due to genetic variations in causative strains and the use of antibiotics, access to broad spectrum and new antibiotics, and difference of climate conditions.

The genes presence in this study demonstrated as following prevalence rates as: lactamase TEM (16.1%), β-lactamase CTXM-3(56.5%), β-lactamase CTXM-2 (8.1%), β-lactamase CTXM-1 (27.4%), β-lactamase SHV (85.5%). Eftekhar et al. in 2012 reported the following prevalence rates: β-lactamase SHV 1.43%), β-lactamase TEM (2.35%), and β-lactamase CTXM (3.31%) in urinary *K. pneumonia* strains [19]. Nasehi et al. reported genes isolation rates of 18% and 26% for β-lactamase TEM and β-lactamase SHV which are lower compared to the present study [20]. In 2010 Faizabadi et al. reported abundance of genes of β-lactamase TEM and β-lactamase SHV as 67.4 and 46.5% respectively (11). The statistics show that Lactamase genes β-lactamase CTXM, β-lactamase SHV, and TEM are increasing and worrying. In most reports frequent Lactamases among *K. pneumonia* isolates was from SHV group derivatives which is consistent with the present study.

## Conclusion

Due to insufficient information about ESBL gene plasmid abundance and genetic pattern in Iran, the diagnosis of *K. pneumonia* strains containing β-lactamase enzyme for better treatment and prevention of the spread of these genes to other bacteria is essential by genotyping and phenotyping methods. However, this study showed that this bacterium is one the most health problem in south west of Iran and more cure should be done for prevention of infection and resistance spread to other area.
